# Measurement of Secondary Structure Changes in Poly-L-lysine and Lysozyme during Acoustically Levitated Single Droplet Drying Experiments by In Situ Raman Spectroscopy

**DOI:** 10.3390/s22031111

**Published:** 2022-02-01

**Authors:** Julian F. A. Perlitz, Lukas Gentner, Phillipp A. B. Braeuer, Stefan Will

**Affiliations:** 1 Lehrstuhl für Technische Thermodynamik (LTT), Friedrich-Alexander-Universität Erlangen-Nürnberg (FAU), D-91058 Erlangen, Germany; julian.perlitz@fau.de (J.F.A.P.); lukas-gentner@t-online.de (L.G.); phillipp.braeuer@fau.de (P.A.B.B.); 2 Erlangen Graduate School in Advanced Optical Technologies (SAOT), Friedrich-Alexander-Universität Erlangen-Nürnberg (FAU), D-91058 Erlangen, Germany

**Keywords:** Raman sensor, in situ Raman spectroscopy, proteins, secondary structure changes, mathematical spectral reconstruction, acoustic levitation

## Abstract

Drying processes such as spray drying, as commonly used in the pharmaceutical industry to convert protein-based drugs into their particulate form, can lead to an irreversible loss of protein activity caused by protein secondary structure changes. Due to the nature of these processes (high droplet number, short drying time), an in situ investigation of the structural changes occurring during a real drying process is hardly possible. Therefore, an approach for the in situ investigation of the expected secondary structural changes during single droplet protein drying in an acoustic levitator by time-resolved Raman spectroscopy was developed and is demonstrated in this paper. For that purpose, a self-developed NIR–Raman sensor generates and detects the Raman signal from the levitated solution droplet. A mathematical spectral reconstruction by multiple Voigt functions is used to quantify the relative secondary structure changes occurring during the drying process. With the developed setup, it was possible to detect and quantify the relative secondary structure changes occurring during single droplet drying experiments for the two chosen model substances: poly-L-lysine, a homopolypeptide widely used as a protein mimic, and lysozyme. Throughout drying, an increase in the β-sheet structure and a decrease in the other two structural elements, α-helix, and random coil, could be identified. In addition, it was observed that the degree of structural changes increased with increasing temperature.

## 1. Introduction

In the pharmaceutical industry, protein-based drugs are often converted into their particulate form by special drying processes such as spray drying in order to improve the stability, shelf life, or application properties [1]. However, these drying and particle formation processes can impose thermal or mechanical stress onto the proteins that may cause undesired changes of the secondary and tertiary (unfolding) protein structure [2]. These structural changes can often lead to irreversible denaturation and loss of enzyme activity [3]. The large number of droplets and the short timescale of these drying and particle formation processes—in the range of milliseconds to seconds—imply major challenges for the time-resolved in situ investigation of the structural changes of proteins [4]. To overcome these challenges, single droplet evaporation and drying experiments have been established in recent years. In general, single droplet experiments can be divided into techniques with or without physical contact of the experimental apparatus to the evaporating droplet. Examples of contact techniques are sessile droplet [5] or pendant droplet methods [6]. Contactless techniques generally require some form of droplet levitation. Among the various existing levitation methods, such as optical [7], diamagnetic [8], electrostatic [9], and aerodynamic levitation [10], acoustic levitation has proven to be particularly suitable for the in situ investigation of single droplet evaporation and particle formation. Here, a liquid sample is levitated in one of the nodal points of a standing acoustic wave, established between an ultrasonic transducer and a reflector. Ultrasonic levitation does not require any specific physical property (e.g., electric charge or magnetism) of the sample, which represents one of the most significant advantages compared to the other methods. Furthermore, it allows for easy access to the sample and features comparable low acquisition and operating costs [11,12]. These properties make acoustic levitation a versatile instrument for physical and chemical analysis. Since the first description of the underlying phenomenon in 1933 [13], the technique has been applied in conjunction with a variety of optical measurement methods, e.g., for the analysis of crystal structure of proteins by X-ray diffraction [14] or the agglomeration of proteins by X-ray scattering [15,16]. For the determination of the geometry, the temperature or species concentrations of levitated samples, techniques such as elastic light scattering, laser-induced fluorescence, and laser-induced phosphorescence have been used [17]. The evaporation behavior and the particle formation process of a single droplet have been investigated by combining acoustic levitation with imaging techniques such as shadowgraphy [18,19] or with absorption techniques such as tunable diode laser absorption spectroscopy (TDLAS) [20]. With techniques such as Fourier transform infrared spectroscopy and UV/V is spectroscopy it is possible to follow the dehydration of particles [21] along with the formation and aggregation of levitated droplets [22]. Furthermore, Raman spectroscopy measurements on levitated samples are another broad field of application. Examples are the investigation of the evaporation behavior and the phase equilibrium of binary mixtures [23,24], the tracking of the dynamics of red blood cells, the detection of hemozoin in malaria-infected cells [25], the identification and environmental monitoring of algal cells [26], and the composition profiling of drying multi-component droplets [27]. In addition to the described applicability to acoustic levitators, Raman spectroscopy is a suitable tool for the qualitative and quantitative analysis of structural elements (in particular the secondary structural elements) of proteins [28]. A large part of the characteristic protein Raman bands originates from the CONH-group. These Raman bands can furthermore be divided into subgroups, known as the amide A band (NH stretching, about 3500 cm^−1^), the amide B band (NH stretching, about 3100 cm^−1^), and the amide I to amide VII bands (I: 1600 cm^−1^–1690 cm^−1^, II: 1480 cm^−1^–1580 cm^−1^, III: 1230 cm^−1^–1320 cm^−1^, IV: 620 cm^−1^–770 cm^−1^, V: 640 cm^−1^–800 cm^−1^, VI: 540 cm^−1^–600 cm^−1^, VII: 200 cm^−1^). Concerning secondary structure, particular interest lies in the amide I and amide III bands, which mainly originate from the C=O stretching vibration (amide I) and from the coupled C-N stretching and N-H bending vibrations (amide III) [29]. Within these two bands, the different secondary structural elements of the proteins, such as α-helix, β-sheet, random coil, and turns, can be characterized by appropriate evaluation methods [30].

Besides Raman spectroscopy, other experimental techniques can be used to determine the protein structure, including nuclear magnetic resonance spectroscopy (NMR) [31], X-ray diffraction (XRD), and X-ray scattering [16,32], as high resolution techniques [33], as well as circular dichroism (CD) and infrared spectroscopy (IR) with main focus on the secondary structure [34,35]. With NMR and CD requiring physical sampling from the investigated process for later ex situ measurements, in situ application of these two techniques for structure determination in a single droplet drying process is not possible. Although in situ application of X-ray scattering at an acoustic levitator has already been demonstrated, X-ray based techniques require high experimental effort and typically special equipment such as a synchrotron [15,16]. Similar to Raman spectroscopy, IR absorption spectroscopy also allows for in situ application and has already been used in combination with acoustic levitation (see above [21]). However, due to the extremely strong and broadband IR absorption of water, IR spectroscopy is problematic for the analysis of aqueous solutions [36]. In contrast to these techniques, Raman spectroscopy completely meets the requirements of a contactless in situ measurement of protein structural changes in aqueous solutions.

The combination of Raman spectroscopy and acoustic levitation of a single droplet enables following the changes in the protein (secondary) structure during the drying process. Compared to conventional methods (measurement in the cuvette and model spray dryer with a subsequent examination of the dried product), this approach offers the advantage of examining the behavior of the protein structure around the critical point of drying, where the formation of a solid protein crust takes place. In the present work, the expected secondary structural changes of the two substances, poly-L-lysine and lysozyme, during single droplet drying and particle formation in an acoustic levitator are assessed by in situ Raman spectroscopy. To that end, a NIR Raman sensor that allows for the excitation and detection of the Raman signal from a levitated (originally aqueous) solution droplet over the whole drying process was developed. The degree of relative structural changes occurring during the drying process is quantified by the ratio of the measured Raman signals of the secondary structural elements, mainly α-helix, β-sheet, and random coil. The signal shares of the different structural components are obtained by a mathematical spectral reconstruction of the Raman spectra with multiple Voigt functions.

## 2. Materials and Methods

### 2.1. Proteins and Sample Preparation

The homopolypeptide poly-L-lysine (purchased as a lyophilized powder from Sigma-Aldrich, now Merck, P2636, LOT # SLBX2635) and the protein lysozyme from chicken egg white (purchased as a lyophilized powder from Sigma-Aldrich, now Merck, L7651, LOT # 072K7062) were used as model substances for the single droplet drying experiments. Poly-L-lysine, the homopolypeptide of the amino acid l-lysine with a molecular weight between 30 and 70 kD was chosen, as its structural conformations can be adjusted as a function of pH and temperature. Additionally, poly-L-lysine shows similarities with the tertiary structure of some fully folded proteins [37,38]. These properties offer the possibility of using poly-l-lysine as a mimic for a protein, as it has already been done in multiple research projects also in connection with Raman spectroscopy [39,40,41]. It should also be noted that in this work, the setting of a specific conformation via the pH value is deliberately omitted. The buffer medium used to set the pH value would itself show several Raman peaks. The latter overlap with the protein peaks, thus impeding the secondary structure quantification by the spectral reconstruction method. Compared to fully folded proteins, it has neither aromatic amino acids nor disulfide bridges, which reduce the number of peaks to be considered in the mathematical spectral reconstruction. In addition, lysozyme was chosen because it is widely used in the study of various drying processes and has also been frequently used in combination with Raman spectroscopy for structural analysis [42,43,44]. The samples were freshly prepared by dissolving a corresponding amount of protein in deionized water to a concentration of 10 wt%. This value of 10 wt% appears as an optimal compromise to ensure a sufficient Raman signal while avoiding potential problems with solubility and handling, which might occur at higher concentrations, the latter being related to droplet injection at higher viscosities. All samples were stored on ice and measured on the same day in the acoustic levitator.

### 2.2. Experimental Setup and Measurement Procedure

**Figure 1:**
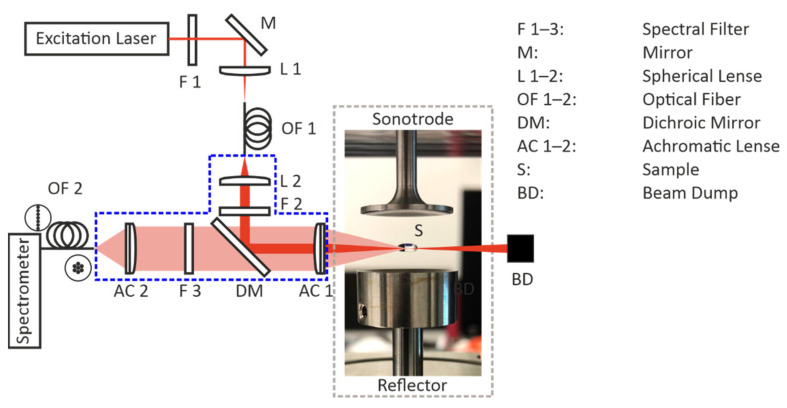
Sketch of the Raman sensor attached to the heating chamber containing the acoustic levitator.

The setup for the single droplet drying experiments consists of two parts: the optical accessible ultrasonic levitator (Borosa Acoustic Levitation L200, Bochum, Germany) and the self-assembled Raman sensor. Figure 1 shows a photograph of a levitated liquid sample in the acoustic levitator and a sketch of the Raman sensor.

The acoustic levitator consists of a sonotrode containing a piezoelectric crystal that emits the ultrasonic wave and a concaved reflector that reflects the wave. If the distance between the sonotrode and the concave reflector is a multiple of half of the wavelength, the acoustic levitator can form a standing wave in between this gap. In the node points of the standing wave nearly any solid or liquid sample can be held in a constant position. The levitator is controlled by a function generator (GW Instek AFG-2005, New-Taipei City, Taiwan) and an amplifier (Keysight Technologies 33502A, Santa Rosa, CA, USA) and levitates the protein solution droplets at a working frequency of around 39 kHz, depending on the drying temperature. The acoustic levitator is surrounded by an optical accessible heating chamber (light gray dotted line in Figure 1) consisting of eight heating cartridges and two type-K thermocouples to adjust a constant drying temperature with an accuracy of ±0.3 °C to ensure drying experiments under defined conditions. For the execution of the Raman experiments, our self-assembled Raman sensor was attached to the acoustic levitator. The Raman sensor itself consists of a laser, the Raman sensor head (marked by the blue dotted line), and a CCD-spectrometers to detect the generated Raman signal. As an excitation source, a continuous-wave laser with a wavelength of 785 nm (Toptica Photonics AG DL pro, München, Germany) was used. The laser wavelength was chosen in order to suppress undesired intrinsic fluorescence signal that occurs in proteins [29]. The excitation light is guided through the adjustable neutral density filter (F 1) to regulate the laser power to prevent damage to the proteins by the laser itself. Next, the spherical lens (L 1) couples the light into an optical fiber (OF 1) with a diameter of 100 µm to guide the light to the Raman sensor head. After leaving the optical fiber, the light is again collimated by a lens (L 2) and passes the laser line filter (F 2) to guarantee a narrow laser line. Afterward, the light reaches the dichroic mirror (DM; Semrock Raman RazorEdge Beamsplitter 785 RU, Rochester, NY, USA), which is highly reflective for the excitation wavelength and transmissive for longer wavelengths. The mirror reflects the beam towards an achromatic lens (AC 1) with a focal length of 60 mm that focuses the laser beam into the droplet. The backscattered signal is collected and collimated by the same achromatic lens (AC 1). The inelastically scattered Raman signal transmits the dichroic mirror (DM) since it is shifted to longer wavelengths with respect to the elastically scattered Rayleigh signal (occurring at the excitation wavelength). Subsequently, a longpass filter (F 3) eliminates the unwanted Rayleigh part of the signal. The remaining Raman signal is focused into a multimode fiber (OF 2) and guided to the CCD-Raman spectrometer (OceanOptics QEpro, Orlando, FL, USA), detecting the inelastically scattered light on 1044 pixels over a wavelength range from 785 to 1150 nm or from 0 to 4000 cm^−1^, respectively. The optical fiber (OF 2) used is a specially manufactured (CeramOptec Handelsgesellschaft mbH, Bonn, Germany) round-to-linear fiber bundle consisting of 15 individual fibers, each 50 µm in diameter (pure fused silica core, 70 µm with cladding). The round end was fused to increase transmission and has a diameter of 240 µm. The linear end exhibits a length of 1.05 mm and is therefore perfectly matched to the height of the entrance slit of the spectrometer (1 mm) ensuring low signal losses. Prior to the Raman measurements, the spectrometer was wavelength-calibrated and the experimental setup was characterized. The wavelength calibration of the spectrometer was performed using a krypton calibration lamp (LOT Quantum Design LSP031, San Diego, CA, USA). The characterization of the wavelength-dependent transmission efficiency of the setup, required for correct reconstruction of the Raman spectra, was performed with a broadband calibration source (OceanOptics HL-3-Plus-CAL, Orlando, FL, USA). For the single droplet drying experiments, which were all carried out at ambient pressure, the levitation cell was set to the desired drying temperature at a constant nitrogen flow rate of 1.0 nL/min. After the manual injection of the protein solution droplet with an initial volume of 4.0 µL by a hydrophobic syringe (SGE syringes, now Trajan Scientific and Medical, 10R-GT-MS1, Ringwood, VIC, Australia), Raman spectra were recorded continuously with an integration time of 15 s per spectrum and a maximum laser power of 70 mW in the focal spot of the laser over the whole drying process. Regarding the reproducibility of the experiments in terms of the initial droplet volume and mass, the accuracy of the volume can be expressed as a value of 1% (stated by the manufacturer of the syringe) whereas the precision of the mass has been gravimetrically determined to be a value of 1.7%. At each temperature, the drying experiment was performed three times.

## 3. Results and Discussion

### 3.1. Drying Protein Droplet Spectra

**Figure 2:**
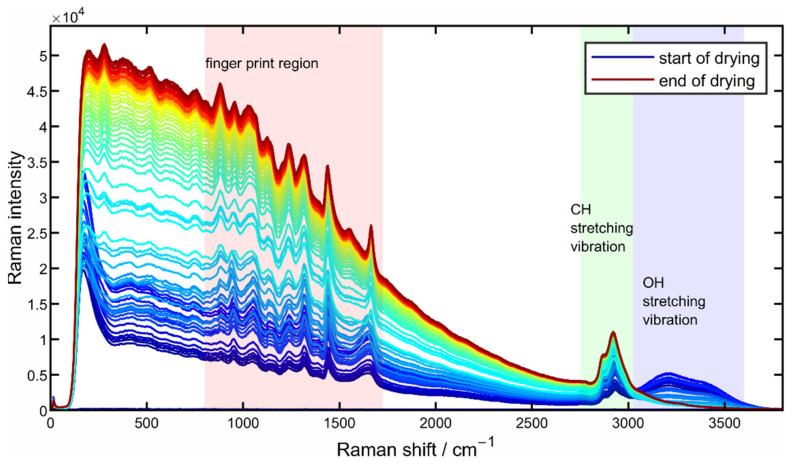
Unprocessed Raman spectrum from the drying process of a 10 wt% poly-L-lysine solution droplet at a drying temperature of 50 °C.

Due to the smaller number of Raman peaks and the resulting clearer presentation, the procedure of spectral evaluation routine is exemplified by a drying experiment of a poly-L-lysine solution drop. Figure 2 shows typical unprocessed Raman spectra from a 10 wt% poly-L-lysine solution droplet recorded over the whole drying process at a drying temperature of 50 °C, where the blue-colored spectrum marks the starting point of the drying process and the red-colored spectrum marks the end of the drying process.

**Figure 3:**
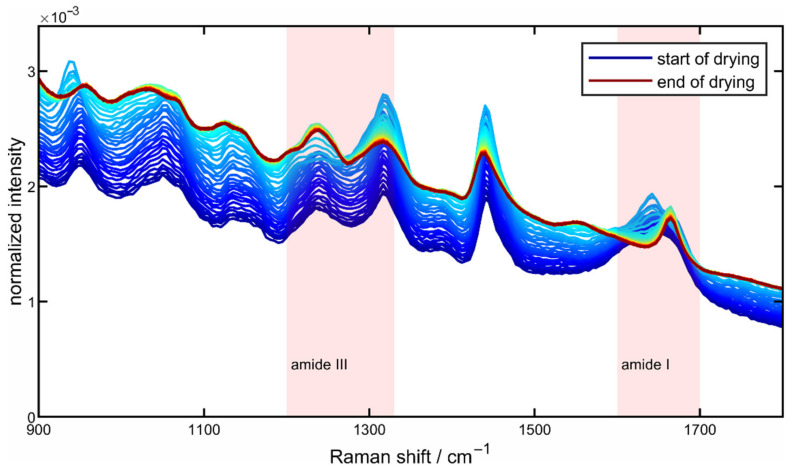
Area normalized fingerprint region between 800 and 1800 cm^−1^ from the drying process of a 10 wt% poly-L-lysine solution droplet at a drying temperature of 50 °C.

The spectrum shown can be roughly divided into three parts, as color-coded in Figure 2: the first part, the area highlighted in blue in the region from 3000 to 3600 cm^−1^, originates from the OH stretching vibration of water. This area of the spectrum can therefore also be used as an indicator of the drying progress as the intensity decreases with the drying time due to the evaporation of water. Directly adjacent to this, an increase in the intensity of the CH stretching oscillation of the protein can be seen in the green-shaded area from about 2700 to 3000 cm^−1^ due to the increasing protein concentration as a result of the evaporation of water. The third area marked in red between about 800 and 1800 cm^−1^ marks the so-called fingerprint region, which is of particular interest with regard to the structural changes of proteins. For a closer look at the spectral changes that occur during drying and for a better description of the regions used for the quantification of the secondary structural changes, Figure 3 shows the fingerprint area in the area-normalized representation.

The two color-coded areas mark the amide I and amide III bands, which can be used to characterize the secondary structure of the proteins. Here, several vibrations are superimposed, which have their origin in the different secondary structural elements and can be assigned to them accordingly [29]. In the present work, a superposition of four vibrations is assumed for the amide I and the amide III bands [45]. The assignments of the four Raman peaks to the corresponding secondary structure elements are summarized in Table 1, presented in the following section. As drying progresses, clear changes can be seen, especially in the amide I band (from 1600 to 1680 cm^−1^), which are expressed in a narrowing of the band and a redshift of the maximum towards larger Raman shifts. In the case of the amide III band (from 1200 to 1300 cm^−1^), clear spectral changes can also be observed over the drying period. Here, a clear change in the peak ratios throughout drying can be seen.

### 3.2. Secondary Structure Shares from Mathematical Reconstruction

In order to quantify secondary protein structure components from Raman spectra, especially from the depicted amide I and amide III bands, multiple methods are described in the literature [46,47]. In the present work, the method proposed by Ngarize et al. [48] is used, calculating the percentage shares of the different secondary structure components (α-helix, β-sheet, and random coil) together with their change over time from the amide bands:

(1) $$x_{i}=\frac{I_{i}}{I_{\mathrm{ref}}}=\frac{I_{i}}{I_{\alpha }+I_{\beta }+I_{\mathrm{rc}}}.$$

Here, the fraction of each secondary structure $x_{i}$ is calculated from the integrated peak intensities of the specific secondary structure $I_{i}$ with respect to their combined intensity $I_{\mathrm{ref}}$ (summation of all integrated peak intensities of the secondary structure components). It should be mentioned that the calculated proportions of the different secondary structures and the changes of these proportions during the drying process in the present work are relative. For a determination of the absolute secondary structure proportions, further measurements would be necessary to determine protein- and vibration-specific quantities such as the scattering cross-section, which is not the main focus of the paper. As the recorded Raman spectra are a combination of several overlapping Raman peaks, a mathematical spectral reconstruction routine was used to obtain the integrated intensities required for the calculation of the secondary structure shares [49]. The Raman peaks are described by Voigt functions (convolution of a Gaussian distribution and Lorentz distribution) which are defined by four parameters: the center peak position $\nu_{s}$, the peak intensity s at the center peak position $\nu_{s}$ and the half widths at half maximum of the Gaussian, $\gamma_{G}$, and the Lorentz distribution, $\gamma_{L}$. For the number and position of the corresponding Raman peaks of the two proteins to be investigated, values from the literature are used [44,50,51]. The calculation of the Voigt functions is based on an approximation proposed by Abrarov and Quine [52,53]. Since there are several other influences on the Raman spectrum besides the Raman peaks of the proteins, it is of great importance to characterize all other influences as far as possible to allow for an undisturbed evaluation of the spectral changes due to structural changes of the proteins. These other influences include the peaks of the solvent (water), a background signal mainly attributable to residual stray light and fluorescence, and the transmission efficiency of the detection system. The signal of water is added to the fit as a so-called hard model or as pre-information, which means that it is always constant in its form and can only be adjusted regarding its intensity. The background signal is generated similarly to the Raman peaks of the proteins by (broadband) Voigt functions and can be adjusted depending on the progress of drying. The transmission properties of the measurement setup are taken into account by the multiplication of the simulated protein and background peaks with the transmission efficiency. This results in the following relationship for the simulated spectrum $a\left(p_{peaks},p_{bg}\right)$:

(2) $$a\left(p_{peaks},\;p_{bg}\right)=\left[VF\left(p_{peaks}\right)+bg\left(p_{bg}\right)\right]\cdot T+hm.$$

Here, the vectors $p_{peaks}$ and $p_{bg}$ include the parameters $\nu_{s}$, s, $\gamma_{G}$, and $\gamma_{L}$ of the Voigt functions of the protein and background peaks, respectively, and the length of these two vectors is defined by the number of Voigt functions to be fitted and the resulting number of parameters. $VF\left(p_{peaks}\right)$ describes the Voigt functions of the protein peaks and $bg\left(p_{bg}\right)$ describes the summation of the (broadband) Voigt functions as background signal depending on the respective parameters. The vector T describes the transmission efficiency and the vector hm describes the influence of the hard model (water). The length of vectors $VF\left(p_{peaks}\right)$, $bg\left(p_{bg}\right)$, T, and hm in Equation (2) is defined by the number of pixels of interest. The fundament of the spectral reconstruction is a weighted nonlinear least-squares regression algorithm with an additional term, inspired by Bayesian regression [49,54]. The aim of the reconstruction is to minimize the residuum $res\left(p_{peaks},p_{bg}\right)$ as a function the parameters $p_{peaks}$ and $p_{bg}$. The residuum is given by:

(3) $$\mathrm{res}\left(p_{peaks},p_{bg}\right)=\overset{n}{\underset{k=1}{\sum }}{\left(\frac{b_{k}-a_{k}\left(p_{peaks},p_{bg}\right)}{\sigma_{\mathrm{noise},k}}\right)}^{2}+\overset{m}{\underset{j=1}{\sum }}{\left(\frac{p_{\mathrm{peaks},j}-p_{\mathrm{peaks}0,j}}{\sigma_{p_{\mathrm{peaks}0,j}}}\right)}^{2}.$$

Here, the first term describes the minimization of the noise-weighted residual ($\sigma_{noise}$) between the measured spectrum b and the simulated spectrum $a\left(p_{peak},p_{bg}\right)$. The length of the vectors $\sigma_{noise}$, b, and $a\left(p_{peak},p_{bg}\right)$ is defined by the number of pixels of interest n. The algorithm also aims at minimizing the residual between the starting parameters $p_{peaks0}$ (prior knowledge) and the actual fit parameters $p_{peaks}$ of the specific protein peaks. The second residual term is weighted by the uncertainties $\sigma_{p_{peaks0}}$ of the respective starting parameters; $p_{peaks0}$, $p_{peaks}$, and $\sigma_{p_{peaks0}}$ are vectors of the length m, where m is defined by the number of Voigt functions to be fitted and the resulting parameters. As starting parameters, e.g., for the center peak positions of the Voigt function, values from the literature are used. The corresponding uncertainties $\sigma_{p}{}_{peaks0}$ arise from the range of the different Raman peak positions (for the same peak) from the literature [29,45,48,55]. This holds true analogously for all parameters to be fitted. Based on these initial parameters and the associated uncertainties, a fit model is obtained for each protein (the values of the starting and the resulting peak positions for the amide I and amide III band are shown in Table 1; it should be emphasized that the values given and the corresponding literature sources only form the basis for the selection of the starting parameters and do not necessarily represent the best or most accurate values.). In general, the more prior information is available and the more accurate it is, the more the corresponding values $\sigma_{p_{peaks0}}$ will decrease. Even without the use of hard boundaries, the provision of such uncertainties will “tie” the results to the starting values, since increasing deviations will lead to larger values of the residuum (see second term in Equation (3)). Such an approach provides high stability of the regression and plausibility of the resulting fit parameters. This regularization is especially important if multiple overlapping peaks (large number of fitting parameters) are present, as the mathematically best fit result might not necessarily be a physically meaningful result.

**Figure 4:**
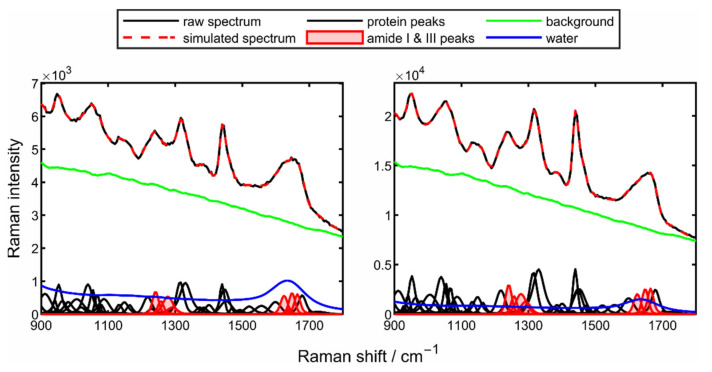
Spectrum of the mathematical reconstruction of the fingerprint region from the drying process of a 10 wt% poly-L-lysine droplet at a drying temperature of 50 °C at the start (**left**) and the end (**right**) of the drying process.

The resulting reconstructed Raman spectra are shown in Figure 4 for the start of the drying process (left) and the end of the drying process (right). For a clearer illustration of the areas relevant for the quantification of the secondary structures, only the reconstructed fingerprint area is shown here.

**Figure 5:**
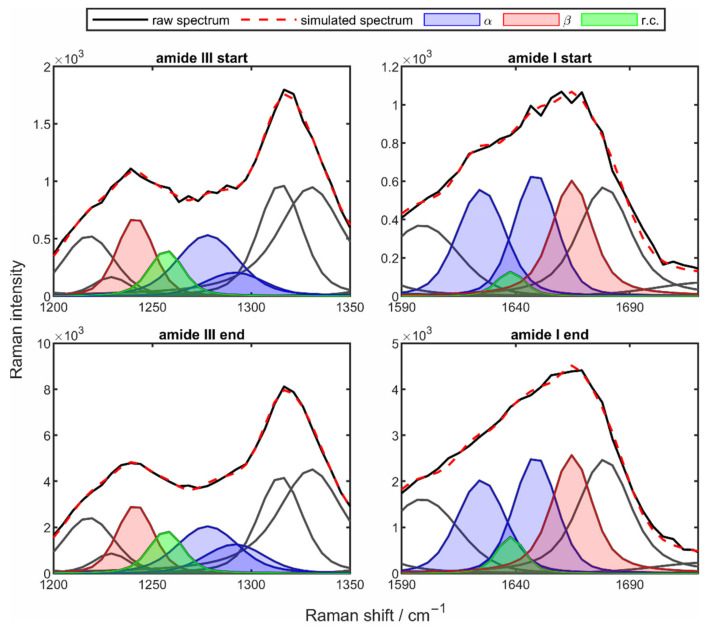
Amide III region (**left column**) and amide I (**right column**) of the reconstructed spectrum from the drying process of a 10 wt% poly-L-lysine solution droplet at a drying temperature of 50 °C at the beginning (**top row**) and the end of the drying process (**bottom row**), with the different secondary structure elements color-coded.

The multiple black Voigt functions – d mark the different protein peaks, where additionally the regions of the amide I and amide III band are highlighted in red. At both points in time, it is visible that the measured spectrum (black line) and the simulated spectrum (red dashed line) are in excellent agreement. It can be seen how the spectrum changes throughout the drying process. Here, the clear influence of water on the Raman spectrum is particularly evident in the region of the amide I band (1600–1680 cm^−1^). Even though minor changes in the peak intensities and peak ratios over the entire spectral range are detectable, the main focus remains on the regions of the two amide bands (red highlighted peaks). Here, significant changes in the peak prominence and thus in the ratio of the peaks can be observed. To illustrate the assignment of the corresponding Raman peaks to the respective secondary structure in the amide I and amide III bands, Figure 5 shows the two bands at the beginning and the end of the drying process.

The two amide bands are described by four peaks each, with the α-helix peaks marked in blue, the β-sheet peaks in red, and the peaks of the random coil structures in green (see legend “α”, “β”, “r.c.”). However, when comparing the two bands, there are some differences in the distribution of the peaks on the different secondary structures. While the β-sheet and the random coil contributions are described by one peak in each band, the α-helix is described by two peaks. When looking at the two amide bands at the different drying times, very clear differences in the strength of the peaks can be observed. For example, in the case of the amide III band (Figure 5, left column), there is a slight increase of β-sheet peaks and random coil peaks at the end of drying (Figure 5, bottom left), whereas one α-helix peak decreases and the other one increases very strongly. In the case of the amide I band (Figure 5, right column), slightly different behavior can be seen. Here, a very strong increase of the β-sheet peak is observed, while all the other peaks decrease sharply during the drying period.

**Table 1:** Assignments of the secondary structure components to the corresponding Raman peaks and overview of the Raman shifts in cm^−1^ of the center peak positions $\nu_{s}$ of the Voigt functions in the amide I and amide III bands of the two investigated proteins poly-L-lysine and lysozyme resulting from the spectral reconstruction with an indication of Raman shifts given in the literature (used as starting parameters).

Secondary Structure	Amide I		Amide III	
	Poly-L-lysine			
	This Work	Literature	This Work	Literature
α-helix	1625	1635 [50]	1278	1276 [41]
	1649	1641 [56]	1292	1305 [57]
β-sheet	1665	1665 [56]	1241	1237 [56]
random coil	1638	1641 [55]	1257	1257 [47]
	lysozyme			
α-helix	1625	1634 [50]	1278	1273 [57]
	1653	1655 [55]	1295	1304 [57]
β-sheet	1667	1669 [58]	1235	1229 [47]
random coil	1635	1639 [55]	1259	1250 [47]

### 3.3. Temporal Changes of the Protein Structure

After the final reconstruction of a whole set of spectra from one droplet drying experiment, the Raman peak intensities of the different secondary structure components are derived from the respective peak parameters, and the relative percentage shares of the secondary structure components can be calculated according to Equation (1).

**Figure 6:**
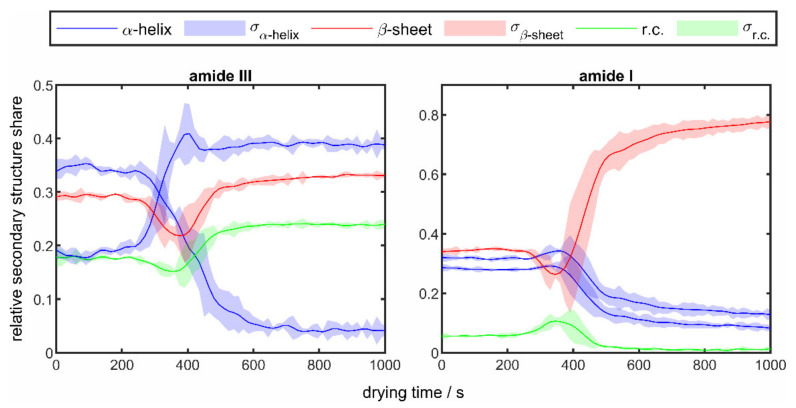
Temporal changes of the relative secondary structure shares in the amide I (**right**) and amide III (**left**) band of 10 wt% poly-L-lysine droplets at a drying temperature of 50 °C.

For poly-L-lysine, Figure 6 shows the changes over time of the secondary structure fractions in the amide I and amide III bands calculated from the changes in the relative peak areas for a drying experiment at a temperature of 50 °C. 

The solid lines show the average values of the structure fractions from the drying of three droplets under identical drying conditions and the surrounding colored areas mark the corresponding standard deviation. In both bands, clear changes can be seen in the course of drying. While the structural components remain at a constant level at the beginning and the end of the drying process, strong changes are seen from a drying time of about 300 s onwards. It is assumed that this point in time corresponds to the critical point of drying, where the transition from a liquid droplet to a solid protein particle takes place. This general drying behavior was also shown in another study by the authors, in which the single droplet evaporation was investigated by means of TDLAS in the drying exhaust air of the levitation chamber [20]. There, lower water concentrations were determined as the protein crust began to form. An increase in the β-sheet fraction can be seen in both amide regions, albeit a much more pronounced one in the region of the amide I band. The behavior of the other structural elements is different. While the fraction of random coil increases slightly in the area of the amide III band, a slight decrease in the area of the amide I band is seen. For α-helix, a decrease for both peaks in the amide I band is observed, whereas in the amide III band one peak increases and the second peak decreases very strongly. In general, the standard deviation also shows a good reproducibility of the results. The increased deviations in the range of the occurring structural changes between 300 and 600 s are mainly due to the levitation process. The levitation parameters, primarily the distance between the sonotrode and the reflector, have a considerable influence on the shape of the droplet and consequently influence the drying speed. For example, a more spherical droplet leads to slower evaporation and formation of the protein crust, thus resulting in a later onset of the structural changes.

**Figure 7:**
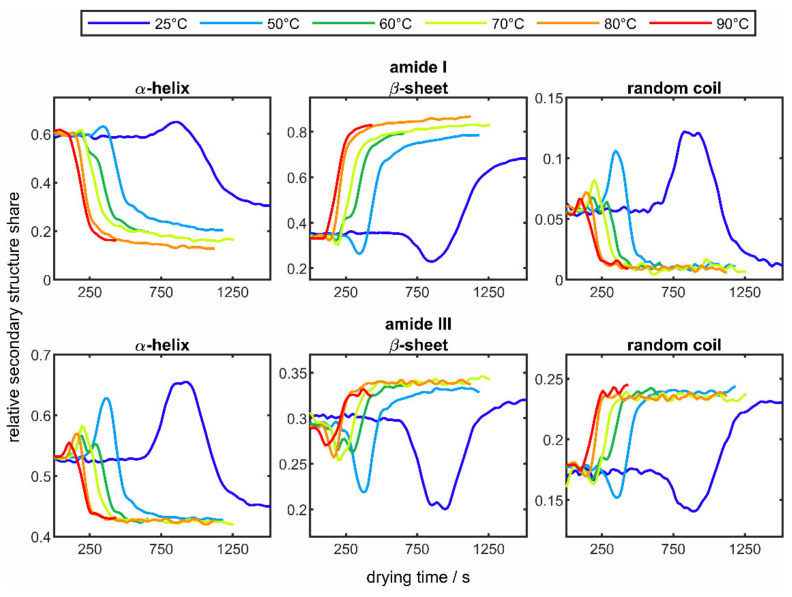
Averaged changes over time of the secondary structure elements α-helix, β-sheet, and random coil of poly-L-lysine in the amide I band (**top row**) and amide III band (**bottom row**) at different drying temperatures between 25 °C (blue line) and 90 °C (red line).

For a clearer presentation, the courses of the three secondary structure elements are shown separately in Figure 7.

In the case of the α-helix, the two individual curves are summarized as one curve. In addition to the known curves at 50 °C, the results of the drying experiments from 25 °C (blue line) to 90 °C (red line) are presented. As in Figure 6, no changes in the three structural elements can be seen at the beginning of each drying process, whereas over the entire drying process there is an increase in the β-sheet structure with a decrease in the α-helix fraction in both amide bands. The described behavior of the change in the secondary structure has already been observed in several works in the literature [57,59,60]. For example, Mauerer and Lee [61] reported an increase in β-sheet structures when spray-drying poly-L-lysine, as these structures are energetically favored in the dried particle. Although the fraction of α-helix is significantly smaller at the end than at the beginning of the experiments, an intermediate increase is observed. This increase in the α-helix fraction could have its origin in the ongoing dehydration, which reduces the stability of the protein. This might be explained by the fact that water molecules and their molecular interactions with the protein chains are of essential importance for the stability of protein structures [62]. As a result of dehydration, poly-L-lysine unfolds, whereby hydrophilic α-helices, which were previously located inside the molecule, are brought to the surface of the protein [63,64]; therefore, the detected fraction of α-helix increases and that of the β-sheet decreases. This assumption is supported when comparing different drying temperatures. Here it can be seen that by raising the drying temperature, resulting in an associated faster dehydration; the protein has less time to unfold and less hydrophilic α-helices are brought to the protein surface. In addition, it can be observed that with elevated temperatures all structural changes occur more quickly and the rate of change increases. For example, in the amide I band, from a temperature of 80 °C onwards, there is no incipient increase any longer. The random coil structure, on the other hand, behaves in the opposite direction in both bands, whereby a faster and more pronounced change can also be seen here at higher temperatures.

**Figure 8:**
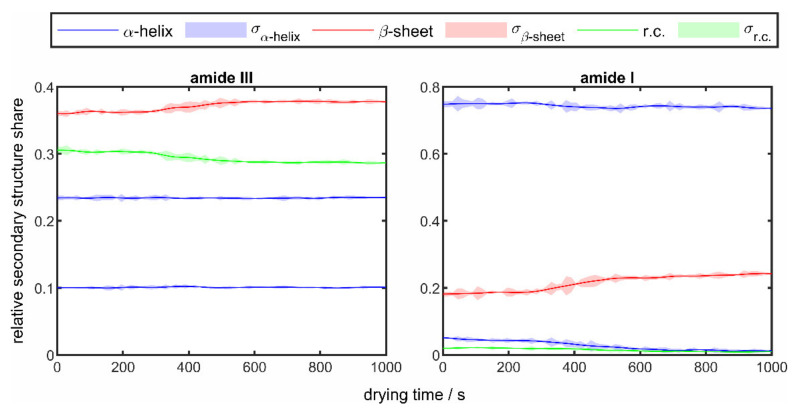
Temporal changes of the relative secondary structure shares in the amide I (**right**) and amide III (**left**) band of 10 wt% lysozyme droplets at a drying temperature of 50 °C.

For lysozyme, the changes over time of the secondary structure fractions in the amide I and amide III bands calculated from the changes in the relative peak areas for a drying experiment at a temperature of 50 °C are shown in Figure 8.

**Figure 9:**
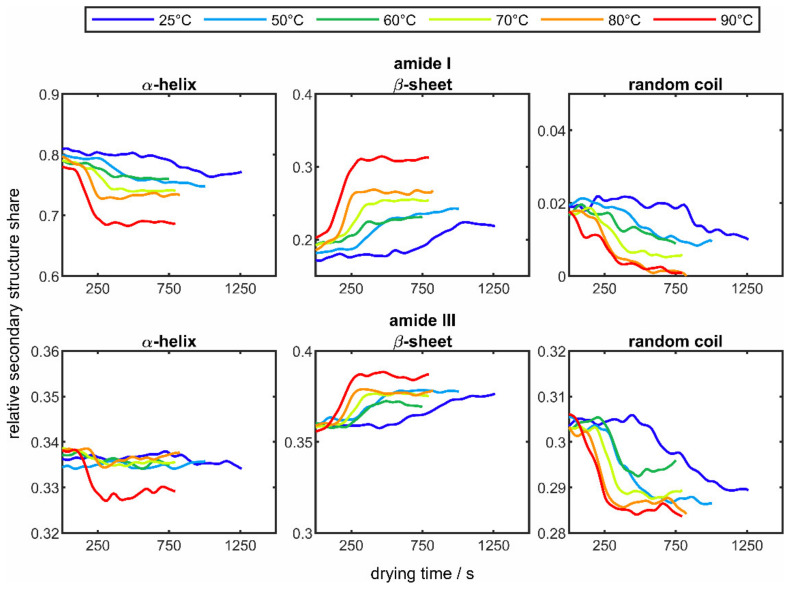
Averaged changes over time of the secondary structure elements α-helix, β-sheet, and random coil of lysozyme in the amide I band (**top row**) and amide III band (**bottom row**) at different drying temperatures between 25 °C (blue line) and 90 °C (red line).

Analogous to Figure 6, the solid lines show the average values of the structure fractions from the drying of three droplets under identical drying conditions and the surrounding colored areas mark the corresponding standard deviations. Compared to the results of the drying experiments of poly-L-lysine in Figure 6, it is noticeable that significantly fewer structural changes occur. It can also be noted that, as with poly-L-lysine, there is a slight increase in the β-sheet structure. In the area of the amide III band, a slight decrease in the random coil structure can be seen, whereas the α-helices remain almost constant throughout the entire drying process. In the area of the amide I band, on the other hand, a slight decrease in the α-helix can also be seen. In order to be able to observe the influence of the drying temperature, the results of the drying experiments of lysozymes at different temperatures in the range of 25 °C (blue line) and 90 °C (red line) are shown in Figure 9, analogous to Figure 7.

When comparing the changes in the secondary structure components in the two amide bands of lysozyme, it can be seen that identical behavior can be observed in both bands, but the degree of change is lower in the amide III band. It is also easily recognizable that the changes occur more quickly with increasing temperature due to faster drying and that the degree of change increases for all structural components. In general, an increase in the β-sheet structure and a decrease in the two other structural elements can be seen throughout drying. The described observation also corresponds to the results of the poly-L-lysine experiments and has already been observed in other studies [65]. The lower degree of structural changes in the drying experiments of lysozyme compared to poly-L-lysine could have its origin in the structure of lysozyme. Disulfide bridges present in the lysozyme can strengthen the stable protein structure [66], which means that the structural changes occurring in the temperature range carried out are less pronounced.

## 4. Conclusions

In this paper, a novel approach of combining the techniques of acoustic levitation and in situ Raman spectroscopy was presented to investigate the relative secondary structure shares of proteins and their changes occurring during the complete drying process of single droplets of protein solutions. To that end, a self-assembled Raman sensor was attached to an optically accessible acoustic levitator, which allows for single droplets to be examined under defined experimental conditions. The homopolypeptide poly-L-lysine and the protein lysozyme were chosen as the substances to be investigated. The relative signal shares of the different secondary structure components α-helix, β-sheet, and random coil and their changes occurring during the single droplet drying process were assessed by a mathematical spectral reconstruction of the detected Raman spectra with multiple Voigt functions. To quantify these relative structural changes, the main focus was on the spectral regions of the amide I and amide III bands. For both substances, an expected increase in the β-sheet structure and a decrease in the α-helix and random coil fractions were confirmed throughout the drying process. In addition, it was observed that the degree of structural changes increases with increasing drying temperature and that the changes occur earlier. To the best of the authors’ knowledge, it was thus possible for the first time to follow the relative changes in the secondary structure of proteins over a complete drying process of a single droplet. Furthermore, a quantification of the relative secondary structure at each point in the drying progress, especially in the period around the formation of a solid protein crust, was accomplished.

The combined use of acoustic levitation and in situ Raman spectroscopy, in conjunction with the chosen method of the mathematical reconstruction of the Raman spectra, provides a suitable method for quantifying the relative secondary structural changes that occur during single droplet drying. This new approach could therefore help to identify the occurrence of irreversible loss of activity of proteins due to structural changes and finally to avoid them in industrial drying processes in pharmaceutical technology.

## Data Availability

The data presented in this study are available on request from the authors.

## References

[1] Maa Y.-F., Prestrelski S.J. (2000). Biopharmaceutical Powders Particle Formation and Formulation Considerations. Curr. Pharm. Biotechnol..

[2] Prestrelski S., Tedeschi N., Arakawa T., Carpenter J. (1993). Dehydration-induced conformational transitions in proteins and their inhibition by stabilizers. Biophys. J..

[3] Branchu S., Forbes R.T., York P., Petrén S., Nyqvist H., Camber O. (1999). Hydroxypropyl-β-cyclodextrin inhibits spray-drying-induced inactivation of β-galactosidase. J. Pharm. Sci..

[4] Cal K., Sollohub K. (2010). Spray Drying Technique. I: Hardware and Process Parameters. J. Pharm. Sci..

[5] Perdana J., Fox M.B., Schutyser M.A.I., Boom R.M. (2011). Single-Droplet Experimentation on Spray Drying: Evaporation of a Sessile Droplet. Chem. Eng. Technol..

[6] Charlesworth D.H., Marshall W.R. (1960). Evaporation from drops containing dissolved solids. AIChE J..

[7] Ashkin A., Dziedzic J.M. (1971). Optical Levitation by Radiation Pressure. Appl. Phys. Lett..

[8] Simon M.D., Geim A.K. (2000). Diamagnetic levitation: Flying frogs and floating magnets (invited). J. Appl. Phys..

[9] Lee S., Wi H.S., Jo W., Cho Y.C., Lee H.H., Jeong S.-Y., Kim Y.-I., Lee G.W. (2016). Multiple pathways of crystal nucleation in an extremely supersaturated aqueous potassium dihydrogen phosphate (KDP) solution droplet. Proc. Natl. Acad. Sci. USA.

[10] Hennet L., Cristiglio V., Kozaily J., Pozdnyakova I., Fischer H.E., Bytchkov A., Drewitt J.W.E., Leydier M., Thiaudière D., Grüner S. (2011). Aerodynamic levitation and laser heating: Applications at synchrotron and neutron sources. Eur. Phys. J. Spec. Top..

[11] Santesson S., Nilsson S. (2004). Airborne chemistry: Acoustic levitation in chemical analysis. Anal. Bioanal. Chem..

[12] Welter E., Neidhart B. (1997). Acoustically levitated droplets—A new tool for micro and trace analysis. Fresenius J. Anal. Bioanal. Chem..

[13] Bücks K., Müller H. (1933). Über einige Beobachtungen an schwingenden Piezoquarzen und ihrem Schallfeld. Zeitschrift Physik.

[14] Tsujino S., Tomizaki T. (2016). Ultrasonic acoustic levitation for fast frame rate X-ray protein crystallography at room temperature. Sci. Rep..

[15] Delißen F., Leiterer J., Bienert R., Emmerling F., Thünemann A.F. (2008). Agglomeration of proteins in acoustically levitated droplets. Anal. Bioanal. Chem..

[16] Leiterer J., Delißen F., Emmerling F., Thünemann A.F., Panne U. (2008). Structure analysis using acoustically levitated droplets. Anal. Bioanal. Chem..

[17] Omrane A., Santesson S., Aldén M., Nilsson S. (2004). Laser techniques in acoustically levitated micro droplets. Lab Chip.

[18] Tuckermann R., Bauerecker S., Neidhart B. (2001). Evaporation rates of alkanes and alkanols from acoustically levitated drops. Anal. Bioanal. Chem..

[19] Kastner O., Brenn G., Rensink D., Tropea C. (2001). The Acoustic Tube Levitator—A Novel Device for Determining the Drying Kinetics of Single Droplets. Chem. Eng. Technol..

[20] Perlitz J., Broß H., Will S. (2021). Measurement of Water Mole Fraction from Acoustically Levitated Pure Water and Protein Water Solution Droplets via Tunable Diode Laser Absorption Spectroscopy (TDLAS) at 1.37 µm. Appl. Sci..

[21] Brotton S.J., Kaiser R.I. (2013). Novel high-temperature and pressure-compatible ultrasonic levitator apparatus coupled to Raman and Fourier transform infrared spectrometers. Rev. Sci. Instrum..

[22] Schenk J., Tröbs L., Emmerling F., Kneipp J., Panne U., Albrecht M. (2012). Simultaneous UV/Vis spectroscopy and surface enhanced Raman scattering of nanoparticle formation and aggregation in levitated droplets. Anal. Methods.

[23] Quiño J., Hellwig T., Griesing M., Pauer W., Moritz H.-U., Will S., Braeuer A. (2015). One-dimensional Raman spectroscopy and shadowgraphy for the analysis of the evaporation behavior of acetone/water drops. Int. J. Heat Mass Transf..

[24] Baer S., Esen C., Ostendorf A. (2014). Phase equilibrium measurements of acoustically levitated squalane-CO_2_ mixtures by Raman spectroscopy. J. Raman Spectrosc..

[25] Puskar L., Tuckermann R., Frosch T., Popp J., Ly V., McNaughton D., Wood B.R. (2007). Raman acoustic levitation spectroscopy of red blood cells and Plasmodium falciparum trophozoites. Lab Chip.

[26] Wood B.R., Heraud P., Stojkovic S., Morrison D., Beardall J., McNaughton D. (2005). A Portable Raman Acoustic Levitation Spectroscopic System for the Identification and Environmental Monitoring of Algal Cells. Anal. Chem..

[27] Lima R.D.S., Braeuer A., Arlabosse P., Ré M.-I. (2020). In situ Raman composition profiling in drying droplets. Powder Technol..

[28] Zölls S., Tantipolphan R., Wiggenhorn M., Winter G., Jiskoot W., Friess W., Hawe A. (2012). Particles in Therapeutic Protein Formulations, Part 1: Overview of Analytical Methods. J. Pharm. Sci..

[29] Rygula A., Majzner K., Marzec K.M., Kaczor A., Pilarczyk M., Baranska M. (2013). Raman spectroscopy of proteins: A review. J. Raman Spectrosc..

[30] Williams R.W., Hirs C.H.W. (1986). Protein secondary structure analysis using Raman amide I and amide III spectra. Enzyme Structure.

[31] Wüthrich K. (1990). Protein structure determination in solution by NMR spectroscopy. J. Biol. Chem..

[32] Ilari A., Savino C., Keith J.M. (2008). Protein Structure Determination by X-Ray Crystallography. Bioinformatics.

[33] Lima D.B., Melchior J.T., Morris J., Barbosa V.C., Chamot-Rooke J., Fioramonte M., Souza T.A.C.B., Fischer J.S.G., Gozzo F.C., Carvalho P.C. (2018). Characterization of homodimer interfaces with cross-linking mass spectrometry and isotopically labeled proteins. Nat. Protoc..

[34] Pelton J.T., McLean L.R. (2000). Spectroscopic Methods for Analysis of Protein Secondary Structure. Anal. Biochem..

[35] Micsonai A., Wien F., Kernya L., Lee Y.-H., Goto Y., Réfrégiers M., Kardos J. (2015). Accurate secondary structure prediction and fold recognition for circular dichroism spectroscopy. Proc. Natl. Acad. Sci. USA.

[36] Schrader B. (1995). Infrared and Raman Spectroscopy: Methods and Applications.

[37] Casier R., Duhamel J. (2020). Effect of Structure on Polypeptide Blobs: A Model Study Using Poly(l-lysine). Langmuir.

[38] Jackson M., Haris P.I., Chapman D. (1989). Conformational transitions in poly(l-lysine): Studies using Fourier transform infrared spectroscopy. BBA Protein Struct. Mol. Enzym..

[39] Mirtič A., Grdadolnik J. (2013). The structure of poly-l-lysine in different solvents. Biophys. Chem..

[40] Kambara O., Tamura A., Naito A., Tominaga K. (2008). Structural changes of poly-l-lysine in solution and lyophilized form. Phys. Chem. Chem. Phys..

[41] Ma L., Ahmed Z., Mikhonin A.A.V., Asher S.A. (2007). UV Resonance Raman Measurements of Poly-l-Lysine’s Conformational Energy Landscapes: Dependence on Perchlorate Concentration and Temperature. J. Phys. Chem. B.

[42] Elkordy A., Forbes R.T., Barry B.W. (2004). Stability of crystallised and spray-dried lysozyme. Int. J. Pharm..

[43] Hedoux A., Ionov R., Willart J.-F., Lerbret A., Affouard F., Guinet Y., Descamps M., Prévost D., Paccou L., Danéde F. (2006). Evidence of a two-stage thermal denaturation process in lysozyme: A Raman scattering and differential scanning calorimetry investigation. J. Chem. Phys..

[44] Dolui S., Mondal A., Roy A., Pal U., Das S., Saha A., Maiti N.C. (2019). Order, Disorder, and Reorder State of Lysozyme: Aggregation Mechanism by Raman Spectroscopy. J. Phys. Chem. B.

[45] Maiti N.C., Apetri M.M., Zagorski M.G., Carey P.R., Anderson V.E. (2004). Raman Spectroscopic Characterization of Secondary Structure in Natively Unfolded Proteins: α-Synuclein. J. Am. Chem. Soc..

[46] Alix A., Pedanou G., Berjot M. (1988). Fast determination of the quantitative secondary structure of proteins by using some parameters of the Raman Amide I band. J. Mol. Struct..

[47] Voicescu M., Ionescu S., Nistor C.L. (2017). Spectroscopic study of 3-Hydroxyflavone-protein interaction in lipidic bi-layers immobilized on silver nanoparticles. Spectrochim. Acta Part A Mol. Biomol. Spectrosc..

[48] Ngarize S., Herman H., Adams A., Howell N. (2004). Comparison of Changes in the Secondary Structure of Unheated, Heated, and High-Pressure-Treated β-Lactoglobulin and Ovalbumin Proteins Using Fourier Transform Raman Spectroscopy and Self-Deconvolution. J. Agric. Food Chem..

[49] Braeuer P.A., Bahr L.A., Röhricht M.-L., Schmidt M., Will S. (2020). Spatially-resolved crystallinity determination of polymer welding seams by Raman-microscopy. Procedia CIRP.

[50] Movasaghi Z., Rehman S., Rehman I.U. (2007). Raman Spectroscopy of Biological Tissues. Appl. Spectrosc. Rev..

[51] Painter P.C., Koenig J.L. (1976). The solution conformation of poly(L-lysine). A Raman and infrared spectroscopic study. Biopolymers.

[52] Abrarov S., Quine B. (2011). Efficient algorithmic implementation of the Voigt/complex error function based on exponential series approximation. Appl. Math. Comput..

[53] Abrarov S.M., Quine B.M. (2012). On the Fourier expansion method for highly accurate computation of the Voigt/complex error function in a rapid algorithm. arXiv.

[54] Bauer F.J., Daun K.J., Huber F.J.T., Will S. (2019). Can soot primary particle size distributions be determined using laser-induced incandescence?. Appl. Phys. A.

[55] Sane S.U., Cramer S.M., Przybycien T.M. (1999). A Holistic Approach to Protein Secondary Structure Characterization Using Amide I Band Raman Spectroscopy. Anal. Biochem..

[56] Carrier D., Pézolet M. (1984). Raman spectroscopic study of the interaction of poly-L-lysine with dipalmitoylphosphatidylglycerol bilayers. Biophys. J..

[57] Herrero A.M. (2008). Raman Spectroscopy for Monitoring Protein Structure in Muscle Food Systems. Crit. Rev. Food Sci. Nutr..

[58] Ye H., Rahul, Kruger U., Wang T., Shi S., Norfleet J., De S. (2019). Burn-Related Collagen Conformational Changes in Ex Vivo Porcine Skin Using Raman Spectroscopy. Sci. Rep..

[59] Czamara K., Petko F., Baranska M., Kaczor A. (2016). Raman microscopy at the subcellular level: A study on early apoptosis in endothelial cells induced by Fas ligand and cycloheximide. Analyst.

[60] Qin Z., Buehler M.J. (2010). Molecular Dynamics Simulation of theα-Helix toβ-Sheet Transition in Coiled Protein Filaments: Evidence for a Critical Filament Length Scale. Phys. Rev. Lett..

[61] Mauerer A., Lee G. (2006). Changes in the amide I FT-IR bands of poly-l-lysine on spray-drying from α-helix, β-sheet or random coil conformations. Eur. J. Pharm. Biopharm..

[62] Mattos C. (2002). Protein–water interactions in a dynamic world. Trends Biochem. Sci..

[63] Haque M.A., Adhikari B., Mujumdar A.S. (2014). Drying and Denaturation of Proteins in Spray Drying Process. Handbook of Industrial Drying.

[64] Martin J., Langer T., Boteva R., Schramel A., Horwich A.L., Hartl F.-U. (1991). Chaperonin-mediated protein folding at the surface of groEL through a “molten globule”-like intermediate. Nature.

[65] Kocherbitov V., Latynis J., Misiūnas A., Barauskas J., Niaura G. (2013). Hydration of Lysozyme Studied by Raman Spectroscopy. J. Phys. Chem. B.

[66] David C., Foley S., Enescu M. (2009). Protein S–S bridge reduction: A Raman and computational study of lysozyme interaction with TCEP. Phys. Chem. Chem. Phys..

